# The perceived usefulness of community based education and service (COBES) regarding students’ rural workplace choices

**DOI:** 10.1186/s12909-016-0650-0

**Published:** 2016-04-29

**Authors:** A. Amalba, W. N. K. A. van Mook, V. Mogre, A. J. J. A. Scherpbier

**Affiliations:** Department of Health Professions Education and Innovative Learning School of Medicine and Health Sciences, University for Development Studies, P. O. Box 1883 Tamale, Ghana; School of Health Professions Education, University of Maastricht, Maastricht, The Netherlands

**Keywords:** Community-based education, Choice of specialty, Rural placement, Medical students, Service, Career choice, Community

## Abstract

**Background:**

Community Based Education and Service (COBES) are those learning activities that make use of the community as a learning environment. COBES exposes students to the public and primary health care needs of rural communities. The purpose of this study was to investigate students’ perceived usefulness of COBES and its potential effect on their choice of career specialty and willingness to work in rural areas.

**Method:**

A mixed method cross sectional study design using semi-structured interviews, questionnaires, and focus group discussions were used for health facility staff, faculty and students and community members.

**Results:**

One hundred and seventy questionnaires were administered to students and 134 were returned (78.8 % response rate). The majority (59.7 %) of students were male. Almost 45 % of the students indicated that COBES will have an influence on their choice of career specialty. An almost equal number (44 %) said COBES will not have an influence on their choice of career specialty. However, 60.3 % of the students perceived that COBES could influence their practice location. More males (64.7 %, *n* = 44) than females (57.8 %, *n* = 26) were likely to indicate that COBES will influence their practice location but the differences were statistically insignificant (*p* = 0.553). The majority of students, who stated that COBES could influence their practice location, said that COBES may influence them to choose to practice in the rural area and that exposure to different disease conditions among different population groups may influence them in their career choice. Other stakeholders held similar views. Qualitative data supported the finding that COBES could influence medical students’ choice of specialty and their practice location.

**Conclusion:**

Medical students’ ‘perceptions of the influence of COBES on their choice of career specialty were varied. However, most of the students felt that COBES could influence them to practice in rural locations.

## Background

Community Based Education and Service (COBES) are those “learning activities that use the community as a learning environment, in which not only students but also teachers, members of the community, and representatives of other sectors are actively engaged throughout the students’ educational experience” [1]. COBES exposes students early in their training and throughout their education to the public health and primary health care needs of rural communities. COBES aims to create awareness among students of the importance of developing community partnerships as a means to implement sustainable healthcare initiatives [2].

Community partnerships are defined as “groups working together with shared goals, responsibilities and power to improve the community” [3]. The community partnerships in COBES include those between community members, governmental and non-governmental organisations, students, faculty members, and health facility staff. By building partnerships between the university, service providers and community as well as the students’ learning and service activities, COBES positively influences and prepares students to care for people in the rural communities [3]. As students work with local, rural health workers and community members, the relevance of COBES and importance of working in rural areas may become internalised as a result of their interaction with these stakeholders in the community [2, 3].

The migration of doctors and other healthcare professionals in Ghana towards the cities and the so-called problem of cross border brain-drain continue to deprive the country of modern health care.

Thus, attention has now been focused on education and retention of medical doctors in Africa. The most commonly reported strategies to improve retention include increasing salaries for faculty, strengthening post-graduate education and launching or strengthening community-based education programmes [4]. There is some evidence that Community- Based Education and Service (COBES) and Problem-Based Learning (PBL) can be used to prepare and acclimatise healthcare professionals to work in rural areas and bring equity in the distribution of health professionals to benefit rural communities [5, 6]. Structured community exposure and community-based education provide students with experiences of working with underserved populations and also improve graduates’ preparation to deal with national health problems [7].

Using the community as a learning environment is compatible with existing learning theories. In this regard COBES can be considered as situated or contextual learning. Contextual or situated learning (also known as situated cognition), or distributed learning refer to situations in which learning and thinking are influenced by the physical and social contexts in which people are immersed. Learning should not be simply viewed as the transmission of abstract and de-contextualised knowledge from one individual to another, but as a social process whereby knowledge is co-constructed.

However, what are stakeholders’ perceptions about the usefulness of COBES for students? Do their attitudes, opinions and associated behaviours have any potential effects on students’ willingness to work in rural areas and thereby influence career choice? Since these aspects have not been subject of prior studies, this study explores the perception of the different stakeholders regarding the usefulness of COBES as a means to attract students to work in rural communities, and influence students’ place of work and choice of specialty.

### Context and methods

#### National context: Ghana

Ghana, located on the West Africa Coast, is a developing country in sub-Sahara Africa with approximately 25 million inhabitants. Africa has only 3 % of the world’s health workforce of 59.2 million, despite bearing 25 % of the global burden of disease [8, 9]. About 70 % of the Ghanaian population live in semi-urban and rural areas (which include most part of Northern Ghana). Ghana is one of the 36 countries in sub-Sahara Africa with a critical shortage of health staff. It has four medical schools which graduate approximately 600 doctors annually [10].

However, despite the graduation of these doctors, national mal-distribution of these, relatively limited number of graduates, migration towards the cities and the so-called problem of cross border brain-drain continue to deprive the country of modern health care [10].

#### Local context, Tamale: COBES

The University for Development Studies, School of Medicine and Health Sciences (UDS-SMHS), established in 1996, is one of the four campuses of UDS and located in Tamale. Tamale is the capital town of the Northern Region, one of the ten regions in Ghana.

In 2007, the UDS-SMHS changed its traditional medical training curriculum to a Problem-Based Learning and Community-Based Education and Service (PBL/COBES) methodology in response to international changes in medical education.

In the COBES component of the PBL/COBES curriculum of UDS-SMHS students are exposed to the community from year one of their medical programme. On yearly basis students spend four weeks in the community with pre-defined objectives until year four. In year five and six, they are scheduled for community posting at district hospitals.

Each of the communities to which the students are sent has a health facility. Students are sent to the selected communities in groups of 10, where they subsequently live, learn and provide service. Each year the students go back to the same community but with different objectives. Currently the UDS-SMHS operates in five (5) districts in the Northern region of Ghana. In each district 3–4 communities are selected for students posting. In addition to the availability of health facilities, communities are selected based on the availability of accommodation and portable water. The presence of electricity in the community is not a pre-requisite but considered a bonus if available. An assigned district supervisor, who is a faculty member, visits these communities to supervise the activities of the students and also meet with staff at the health facilities, the chief and opinion leaders of the community,

## Methods

### Participants

The participants of the study included community members, health facility staff, lecturers and medical students as stakeholders of COBES. Inclusion criteria for community members were being older than 18 years of age, being an opinion leader (assemblymen who are the local political leaders for the communities, youth leaders, women leaders’ advocates and religious leaders) and having lived in the community for the immediate past 5 years. Medical students who have been to the community for at least two COBES sessions of 4 weeks duration for two consecutive years were eligible to participate. Only medical students in medical year three and four met the inclusion criteria. Medical year two was excluded from the study since they had been to the community only once. From medical year five, students are sent to the district hospitals for COBES which has a different focus. Health facility staff who have worked and interacted with students at the facility level for at least one year were also eligible to participate in the study. A multistage random sampling process was used to select four (4) districts and then two communities (from each of the four districts). UDS-SMHS operates in five (5) districts in the Northern region of Ghana. In each district, 3–4 communities have been selected for students posting based on agreed criteria. For the purposes of this study we randomly selected 4 districts through the use of lottery. After selecting the four districts, a similar simple random process was adopted again to select 2 communities from each of the randomly selected districts.

Lecturers with prior involvement in COBES activities were qualified to participate in the study. Lecturers were considered to have been involved in COBES if they had supervised students as district coordinators or participated in the assessments of students during COBES. The purpose of the study was clearly explained to all participants (i.e., community members, health worker, students and lecturers) and informed consent was obtained before participation. Translation of interview guide into the local dialect was done for community members who could neither speak nor write in the English Language. Participation in the study was voluntary and confidentiality and anonymity were ensured. Participants were reimbursed the cost of transportation and lunch was provided as a form of incentive. Ethical approval was granted by the Ethics Committee of the School of Medicine and Health Sciences, University for Development Studies.

### Data collection tools

This study adopted open-ended questionnaires, focus group discussions and key informant interviews to collect data.

#### Questionnaire

Data from 10 lecturers and 134 students was obtained using nine open-ended questions with ample space for narrative comments. The demographic variables age and gender were included in the questionnaires (see Appendix 1). Items of the questionnaire and the focus group discussion guide were derived from review of the literature and through discussions among the authors and other relevant subject area experts. The subject area experts reviewed the items for content validity. The items were piloted on a sample of ten participants (5 from each year group) to ensure that they were comprehensible by the study participants. There were minor changes in the form of editing the English. These ten participants did not take part in the study.

#### Focus group discussions

Focus group discussions (see Appendix 2) were used for the community members to collect shared understanding and collective opinions or views on a particular issue rather than individual views. In all 16 focus group discussions were undertaken (8 for male and 8 for female) and each had duration of about 90 min. Each focus group had 8 members with each group having either only females or males. This was done to enable women feel comfortable to express their opinions. Culturally, women do not engage in debates with men on contentious issues at the community level. Discussants were selected through purposive sampling. With the aid of a discussion guide, all discussions were done by four trained research assistants having prior experience in conducting focus group discussions. During discussions, the research assistant probed further for either details of information or clarification of issues for better understanding

#### Key informant interview

Using an interview guide (see Appendix 3), key informant interviews were used for health facility staff to get their opinion regarding the relevance of COBES to them and how they think COBES could influence the practice location of medical students. These interviews were carried out by trained research assistants. Using an interview guide the interviewer also probed further for more detailed information.

### Data analysis

All quantitative data was analyzed using Statistical Package for Social Sciences (SPSS) version 18 (SPSS Inc, IBM, Chicago, IL, USA) Descriptive statistics of frequencies and percentages were used to describe the data. Data was compared using Fisher’s exact test at *p*-value of <0.05 considered statistically significant.

All interviews and discussions were audiotaped and transcribed verbatim. Data analysis was done in phases according to generally accepted coding principles including open coding, axial coding and selective coding (Cohen, 2007). The initial coding and development of categories was done by the first author (AA). Coding was checked by the third author (VM) who has had some training in qualitative research. The first and second authors (AA, WvM) thereafter checked the coding and resulting categories. Any discrepancies in the process were discussed until consensus was reached.

## Results

This section consecutively discusses the numerical results of the questionnaires, the results of the qualitative analysis of the focus group discussion (FGD), questionnaire and guided interviews (see Appendices 2 and 3 respectively). The qualitative results from the separate research tools are thereafter aggregated due to revealed overlap of the results. The resulting main overall results are presented in subsequent sections. Appropriate quotes from the different groups are cited to illustrate the main themes.

### Quantitative results of the student questionnaire

From the 170 questionnaires administered, 134 were returned (78.8 % response rate). Majority (59.7 %) were male. 59 and 41 % of the students were in medical year 3 and 4 respectively (see Table 2). Thirty and 17 students in years 3 and 4 respectively did not specifically answer the questions on: “will your experience in the community through COBES affect your choice of specialty’?” And ‘”how will your experience in the community through COBES affect your choice of practice location?”’ These were considered as missing values and therefore were not used in the analysis of these two specific questions. They however answered other questions in the questionnaire that addressed other aspects of the research question. Almost 45 % of the students indicated that COBES will have an influence on their choice of career specialty, while an almost equal number (44 %) said it will not have an influence on their choice of career specialty (shown in Table 1). However, 60.3 % of the students perceived that COBES could influence their practice location. Although a high proportion of females indicated that COBES could not influence their choice of specialty, the differences were not statistically significant using Fisher’s exact test. More males (64.7 %, *n* = 44) than females (57.8 %, *n* = 26) were more likely to indicate that COBES will influence their practice location but the differences were statistically insignificant (*p* = 0.553).

**Table 1:** Students’ reported perceived influence of COBES on choice of career specialty and practice location stratified by gender

Variable	Total	Male	Females	*p*-value
Choice of career specialty	(*n* = 105)	(*n* = 65)	(*n* = 40)	
Influences	47(44.8 %)	29(44.6 %)	18(45.0 %)	1.000
Does not influence	46(43.8 %)	29(44.6 %)	17(37.8 %)	0.843
Uncertain	12(11.4 %)	7(10.8 %)	5(11.1 %)	0.764
Missing (*n* = 29)		(15)	(14)	
Practice location	Total (*n* = 116)	(*n* = 68)	(*n* = 45)	
Influences	70(60.3 %)	44(64.7 %)	26(57.8 %)	0.553
Does not influence	41(35.3 %)	24(35.3 %)	17(37.8 %)	0.694
Uncertain	5(4.3 %)	3(4.4 %)	2(4.4 %)	1.000
Missing (*n* = 18)		(9)	(9)	

Shown in Table 2 are students’ perceived influence of COBES on their choice of specialty and practice location, stratified by level of medical training. Significantly, students in medical year three were more likely than their counterparts in medical year four to report that COBES will influence their choice of career specialty (54.2 % vs. 32.6 %; *p* = 0.031) and practice locations (69.6 % vs. 46.8 %; *p* = 0.020).

**Table 2:** Students’ reported perceived influence of COBES on choice career specialty and practice location stratified by level of medical training

Variable	Medical year three (*n* = 79)	Medical year four (*n* = 55)	*p*-value
Choice of career specialty	(*n* = 59)	(*n* = 46)	
Influences	32(54.2 %)	15(32.6 %)	0.031
Does not influence	23(39.0 %)	23(50.0 %)	0.323
Uncertain	4(6.8 %)	8(17.4 %)	0.124
Missing (*n* = 29)	(20)	(9)	
Practice location	(*n* = 69)	(*n* = 47)	
Influences	48(69.6 %)	22(46.8 %)	0.020
Does not influence	18(26.1 %)	23(49.0 %)	0.017
Uncertain	3(5.1 %)	2(4.3 %)	1.000
Missing (*n* = 18)	(10)	(8)	

Although the results are presented in an aggregated way due to large commonalities in response, there was one peculiar difference in response that came solely from the health facility staff in terms of support for training.

### Qualitative results

The qualitative results of the Focus Group Discussions, questionnaires and guided interview analysis are presented based on subjects/themes that were identified from the different research techniques used. Consecutively, how COBES is perceived to influence choice of specialty and practice location, benefits of COBES to the community and to the students will be discussed, finalizing with suggested future improvement of COBES. Illustrative quotes are provided in italics.

### How COBES could influence students’ choice of specialty

Participants indicated that the decision to make a choice on which areas students specialise is influenced by various factors including: the awareness of the needs of the community, the inadequacy (both in numbers and levels of professional training) of healthcare personnel or doctors in the rural community, the right of the rural person to healthcare and the exposure to different fields of medicine. It becomes clear that the community’s lack of basic access to healthcare may motivate some of the students to want to fill this gap after their graduation. As shown in the results in Table 1, almost 45 % of the students indicated that COBES will affect their choice of specialty with more males indicating so.

‘*Most surgical patients have to be referred to Bole (district hospital), and patients lose their lives when the only surgeon in Bole is busy or is not around, hence I will like to be a surgeon’. (Male student)*

Early patient contact during COBES exposes the students to various disease conditions, and also allows them to work under various health professionals in the community. They get the chance to practice some of the skills learnt in the skills laboratory at the faculty on real patients. This gives them the opportunity to identify areas of interest to want to specialise.

*‘I got the opportunity to assist in delivery of a baby and that gave me an interest in O & G’ (Female student)*

### How COBES could influence students’ practice location

The willingness and preference of students to work in the rural area is not only due to their mere presence in the community but also due to awareness of community health needs and the limited human resources. These are pull factors for students to want to work in the rural area.

*‘The communities have so many health problems with less experienced health professionals. So I will prefer to work closer to these communities’ (Male student).*

The student’s adaptation and adjustment to rural lifestyles make them to cope with living in the rural community. COBES develops students to adapt to rural lifestyle making it easy to accept to work in rural communities. This is evident by the following quotes.

*‘If I am able to stand the challenges during COBES, I don’t think there will be any other community I can’t work in or survive. Hence, I will accept posting anywhere’ (Female student).*

*‘Through COBES students are already exposed to the rural communities. They may appreciate the settings and thus accept rural postings as practice location’ (A lecturer).*

During COBES the students realise the need for equity in health care; that health is not the preserve of the affluent in the cities but that the poor rural people also have the right to quality healthcare.

‘COBES *makes me realise that the poor also needs better health, has encouraged me to work to reach out to the poor and needy in the way I can’ (Male student).*

The welcoming reception and hospitality the community members’ accorded to the students may be a motivating factor for graduates to go back to the community to practice especially when the students see the societal benefits in preventing diseases in the community. This is made obvious by the following quote.

‘*I would gladly accept posting to my COBES community or a similar community due to the warm reception received and the hospitality of the people’ (Male students).*

### Benefits of COBES to the community

The study participants cited a number of benefits to the community.

#### Health education and promotion

The community sees the relevance of health promotion and education. As the students give talks on health education and carry out health promotion activities, the behaviour of the community, as well as their health seeking behaviour changes and their awareness towards health and their knowledge on health issues improve.

*‘It has improved their way of living especially health-wise. Pregnant women have also known the importance of antenatal and postnatal care’ (Female student).*

#### Serves as role model to the youth

The presence of the students in the community serves as motivation for the youth. In the northern part of the country where most families do not see the relevance of children’s education, parents do not invest in their education. Encountering female students may convince parents that educating the female offspring can be very rewarding, as evidenced by the following quotes.

‘*Motivates the young ones in the community to take their studies more serious since they may see them(students) as role models and therefore aspire to be like them’ (Lecturer).*

#### Service to the community

Participants indicated that the presence of students in the community provides workforce to the community. They are also able to identify the needs of the community and propose solutions with the support of the community members.

*‘Students solve basic community problems. Students provide services to the community. Members of the community learn positive lifestyle from the students’ (Male student).*

### Benefits of COBES to the students

Participants alluded to the fact that students do benefit a lot from COBES activities in the communities. Stakeholders acknowledged that the community serves as a learning platform where students interact with people of different cultural backgrounds. This helps them improve their communication skills, help to build their clinical and social skills and empowers them in their clinical work. COBES helps students get a clear understanding of primary health care setting within the health structure. Having part of their training in the community helps them to make choices as to which areas they want to specialise and also develop interest to practice in the rural area after graduation.

*‘As a result of COBES some students develop on interest in practicing in rural areas after graduation’ (Male student).*

*‘When in school they only learn what is in the book but when they come here they can apply what they have learnt and by this they gain more experience. They see diseases and symptoms in books but as they come here to see people with these health conditions in the community and how these illnesses have changed the physical outlook of these people. He or she can observe the clients much better than as learned in the books. This can enhance their knowledge’ (A community youth leader).*

### Training support from University

Some of the health facility staff were of the opinion that since most of those who guide the students in the community are community health nurses, the University, as a way of incentive, could offer some of them admission into the University to pursue further studies to better guide the students who are more knowledgeable than them.

*‘Though the community health nurses have some experience, we will need further training in the University to better guide the students,-------otherwise the way it is instead of we guiding the students, they would be teaching us’ (Community health nurse).*

### Suggestions to improve the organisation of COBES in the future

Participants indicated a number of ‘push factors’ that if addressed would go a long way to improve the organisation of COBES to increase students interest of practicing in rural communities when they graduate. Students suggested that both past and present students should be involved in the planning committee of COBES for better organisation of the programme. The lack of basic equipment at the facility level was de-motivating and called for improvement of the poorly equipped health facilities. It was suggested that intervention proposal written by the students and the community should be made available to the NGOs and District Assemblies to solicit for funds to address some of the identified community needs so as to improve their social amenities. Staff and lecturers should be motivated to spend adequate number of days in guiding the students in the communities.

## Discussion

This study revealed that about 45 % of the students indicated that COBES will influence their choice of specialty. Though an equal number had a contrary view of the effect of COBES on their choice of specialty, educational experiences in the community may influence the choice of specialty of medical students. As shown in the study, students cited a number of reasons why COBES could influence their choice of specialty. Being confronted with the needs of the community such as inadequate health professionals, different disease conditions as well as different specialities of medicine were some of the reasons observed by students. These experiences may motivate students to choose certain areas of medicine to specialise in. A desire for rural practice is an important factor in the choice of rural primary care as a career [11]. It is however, noteworthy that about 44 % of the students did indicate that COBES will not influence their choice of specialty. This probably suggests that students may not be satisfied with certain aspects of the COBES program. As suggested by the students and other stakeholders, structural improvement in the organisation of COBES such as improved transport arrangements, accommodation, equipped facilities, the presence of doctors may influence the decision regarding the choice of specialty and practice location of such groups in future.

An important finding of this study was that majority of the students (60.3 % vs. 35.3 %) said their experiences during COBES may influence their practice location and as evidenced by our qualitative data, most of these students indicated their willingness to practice in rural areas. This is supported by similar studies in South Africa [12] and Uganda [2] which indicated that the decision to ‘go rural’ is not automatic, but is seemingly facilitated by other factors such as awareness of the needs of the rural area, role modelling, and exposure to rural training. The importance of this finding is that there is some evidence that Community- Based Education and Service (COBES) can be used to prepare and acclimatise healthcare professionals to work in rural areas and bring equity in the distribution of health professionals to benefit rural communities [5, 6].

The Ghana Ministry of Health (MoH) has implemented a number of incentives aimed at limiting the migration of doctors and other health professionals in Ghana towards the cities and the so-called problem of cross border brain-drain which continue to deprive the country of modern health care [10]. These incentives included a 20–30 % salary top up for health staff in deprived areas (implemented in 2004) and a staff vehicle purchase scheme (implemented in 1997) [13]. However, neither has yielded the desired results in addressing the lack of health professionals in remote areas. Wilson and Couper [14] described coercion as forced redress where penalties are applied if doctors do not comply with certain requirements like ‘community service,’ requirement to register as a doctor, rural experience required prior to further specialisation and limiting foreign health professional recruitment to rural practice [14]. In Ghana however, rural experience as a requirement prior to registration as a medical doctor or further specialisation is not a requirement.

Therefore, attention must be focused on structured community exposure and community-based education to provide students with experiences working with underserved populations and improve graduates’ preparation to deal with national health problems [7]. A sense of social responsibility develops among the students as they interact with community members.

Using the rural community as a platform to prepare graduates to work in rural communities is consistent with similar studies in Uganda [15], Canada, and Australia [16].

A positive rural practice experience during medical school can positively influence students’ attitudes towards rural practice and eventual practice location in rural areas [17–22]. According to Kaufman et al. [23] rural training sites are ideal locations for students to confront the array of social, political and economic forces underlying ill health in our society. Exposing students to an environment that ‘typically resembles’ what students will encounter in later professional life helps them to be acclimatised to the harsh conditions which builds them up to face future challenges and also changes their mind-set of community life. They see the community as a learning platform which is compatible with existing learning theories that is contextual or situated learning.

**Fig. 1:**
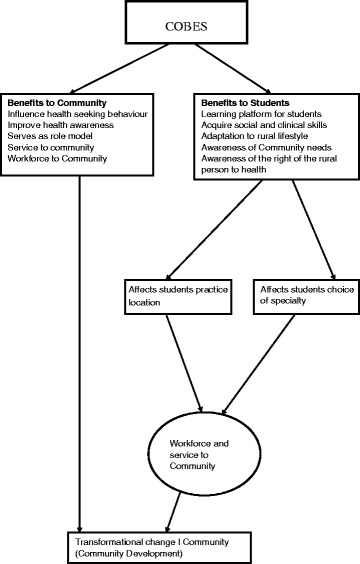
Benefits of COBES

As shown in the model diagram (see Fig. 1) continuous presence of students in the community brings a lot of benefits. These include improved quality of health services, helping to carry out community projects, serving as role models to the youth. This eventually leads to community development/transformation.

Transformative learning derived from the works of education theorists, notably those of Freire [24] and Mezirow [25] have three successive levels-moving from informative to formative to transformative learning. During COBES, students start from the informative level by acquiring knowledge and skills. Through socialising students around values and attitudes they move to the level of formative learning. Finally, students move to transformative learning when students develop leadership attributes with the aim of making them become enlightened change agents [26].

A welcoming reception and hospitality by the community members’ may be a motivating factor for graduates to go back to the community to practice. This may be influenced by students realizing the social benefits of preventing diseases in the community. This has been similarly reported by Couper et al. [27], in South Africa in which it was revealed that a close relationship with the community, appreciation and a sense of acceptance by the community are reinforcing factors for graduates to choose rural placement. It is important to identify and encourage role models in the rural community as they have greater influence on career choice in primary health care and this is an important principle in future health professional provision. The importance of role models in influencing career choice has been similarly reported in other studies in sub-Sahara Africa [27].

Another important finding of this study was that, students in medical year three were more likely than students in medical year four to say that COBES may influence their choice of practice location. This is unexpected, as we anticipated students to develop more interests to working in rural communities as they progress in the program. Probably students in medical year four observed that the current form of the COBES program may not be meeting its objectives and may have to be revised. Students observed several challenges with the current form of COBES including poorly equipped health facilities and inadequate involvement of past students, among others. It’s imperative to note that the COBES program is currently undergoing a review to take care of the challenges identified by the students and other relevant stakeholders.

Despite the challenges cited by students and other stakeholders in this study, both students and community members expressed satisfaction with the programme and its likelihood of influencing students to work in the rural area after graduation. This has been similarly reported in other studies [28, 29].

For future improvement of the COBES programme, students, community members as well as lecturers identified a number of educational and social challenges such as: lack of professional development, poorly equipped health facilities, lack of accommodation, bad road network and inadequate transport system to move within the community, which if adequately addressed would provide a more enabling learning educational environment. The University could, for example, lower the entry requirement for admission for the community health nurses, who guide the students, to further their education as a way of incentive. The challenges or barriers which mitigate against students practice location after graduation and as such serves as major barriers to healthcare capacity building is not limited to Ghana alone but these barriers have been similarly reported in studies across sub-Sahara Africa [4, 7, 28]. The Ghana Ministry of Health (MOH) and Ministry of Education (MOE) could begin to initiate pilot interventions especially educational reforms such as launching or strengthening COBES aimed at improving retention of health workers in deprived/hardship areas based on available evidence.

### Limitations

The present study has limitations. The students who participated in the study were third and fourth year students who had participated in COBES activities at least for two consecutive years. Their views and perceptions may change as they progress along the academic career and after graduation. The views of the graduates were not included in this study.

The study was carried out by staff of the School of Medicine and Health Sciences, most of who have the passion and positive attitude towards COBES and this might have created some bias. However, an attempt to prevent this was our use of a questionnaire with open ended questions and probing into rationales where necessary. Furthermore, the survey was conducted in a single Ghanaian medical school, making it difficult to generalize our findings. However, the findings are in agreement with similar studies conducted in both developed and developing countries. It also serves as a baseline for further studies in other medical schools in Ghana.

## Conclusion

Medical students ‘perceptions of the influence of COBES on their choice of career specialty were varied. Most of the students however, felt that COBES could influence them to practice in rural locations. Health facility staff, faculty and community members applauded the COBES programme and generally indicated that the COBES programme could encourage graduates to choose rural places to work if a holistic supportive learning environment was provided for the students.

Almost equal proportions of students perceived that COBES could influence their choice of career specialty and practice location. Being aware of the needs of the community such as inadequate doctors or health professionals and the right of the community members to have equal access to health were some of the push factors that could influence students’ choice of specialty and practice location. Students’ being able to adapt to rural lifestyle through COBES may also be important in influencing students’ choice of career specialty and practice location.

The findings of this study suggests that using the community as a training environment may help to address the inequality or mal-distributions of doctors and other health professionals in the country and other Sub-Saharan African countries in similar circumstances. Future research could be conducted to explore the reasons why students think COBES will not influence their practice location or choice of specialty in order to guide further improvement of the COBES curriculum.

## Availability of data and materials

All the data and additional supporting files on which the conclusions of this manuscript rely are available without restrictions by contacting the corresponding author.

## Appendix 1: Students- questionnaire

### Introduction

The School of Medicine and Health Sciences (SMHS) of the University for Development Studies (UDS), Ghana, successfully adopted its traditional medical training curriculum to Problem-Based Learning (PBL) and Community-Based Education and Service (COBES) methodology in 2007. COBES describes those “learning activities that use the community extensively as a learning environment, in which not only students but also teachers, members of the community, and representatives of other sectors are actively engaged throughout the educational experience” (World Health Organization, WHO, 1987). COBES component of the PBL/COBES curriculum of UDS-SMHS is the process by which teaching and learning is done in the community. Students start community exposure from year one of the medical programme and spend four weeks in community and this continues on a yearly bases where the students go back to the same community with defined objectives

Demographic characteristics

Age:

Gender:

Level:

1. How many years have you participated in COBES at UDS-SMHS?
2. What are the opportunities available to students going on rural placement?
3. What would in your opinion encourage students to want to choose a rural placement?
4. What are some of the challenges that you faced during COBES?
5. How will your experience in the community through COBES affect your choice of **SPECIALTY?**
6. How will your experience in the community through COBES affect your practice **LOCATION?**
7. How will you describe the benefits of COBES to the community?
8. In what way do you think COBES can be used to develop the interest of graduates to work in the rural area?
9. What would you suggest as a way of improving the COBES activities that will positively influence a rural workplace after graduation

## Appendix 2: Community-focus group discussion guide

### Introduction

The School of Medicine and Health Sciences (SMHS) of the University for Development Studies (UDS), Ghana, successfully adopted its traditional medical training curriculum to Problem-Based Learning (PBL) and Community-Based Education and Service (COBES) methodology in 2007. COBES describes those “learning activities that use the community extensively as a learning environment, in which not only students but also teachers, members of the community, and representatives of other sectors are actively engaged throughout the educational experience” (World Health Organization, WHO, 1987). COBES component of the PBL/COBES curriculum of UDS-SMHS is the process by which teaching and learning is done in the community. Students start community exposure from year one of the medical programme and spend four weeks in community and this continues on a yearly bases where the students go back to the same community with defined objectives

1. What do you think are some of the factors that will encourage trainees to work in rural communities (personal and community factors)
2. In your opinion, how beneficial is COBES to trainees and rural communities
3. What are the challenges you encountered as a result of the students stay in your community?
4. What will you do as a community to encourage students to come back to your community after graduation?
5. What, in your opinion are the challenges faced by students during their stay in the community?
6. How can COBES be improved for the benefit of trainees and the community
7. Do you think the PBL with COBES has any influence on trainees choice of place of work and how does it influence this choice.

## Appendix 3: Health workers/district hospitals- interview guide

1. What would in your opinion encourage students to want to choose a rural placement
2. What do you think would be additional opportunities and benefits of students going on rural placement
3. In what way do you think COBES can be used to improve the interest of graduates to work in the rural area?
4. What would you suggest as a way of improving the COBES activities the will positively influence a rural workplace after graduation
5. What will you do as a facility to encourage students to come back to your facility after graduation
6. What are in your opinion the challenges faced by students during their stay in their communities

## Abbreviations

COBES: Community-Based Education and Service
MOE: Ministry of Education
MOH: Ministry of Health
PBL: Problem-Based Learning
SMHS: School of Medicine and Health Sciences
UDS: University for Development Studies
